# Age-Related Changes in Children’s Associations of Economic Resources and Race

**DOI:** 10.3389/fpsyg.2016.00884

**Published:** 2016-06-16

**Authors:** Laura Elenbaas, Melanie Killen

**Affiliations:** Department of Human Development and Quantitative Methodology, University of MarylandCollege Park, MD, USA

**Keywords:** social cognition, social cognitive development, developmental intergroup relations, social status

## Abstract

Age-related changes in children’s associations of economic resources and race were investigated. The sample (*N* = 308) included 5–6 year-olds (*n* = 153, *M* = 6.01 years, *SD* = 0.33 years) and 10–11 year-olds (*n* = 155, *M* = 11.12 years, *SD* = 0.59 years) of African–American (*n* = 93), European–American (*n* = 92), Latino (*n* = 62), Asian–American (*n* = 23), and multi-racial or multi-ethnic (*n* = 26) background. Participants matched pairs of target children (African–American and European–American) with visual indicators of low, middle, and high economic status. Children’s associations of economic resources with racial groups changed with age, and reflected different associations at high, middle, and low levels of the economic spectrum. Specifically, children associated targets of both races with middle economic status at a comparable rate, and with age, increasingly associated targets of both races with indicators of middle economic status. By contrast, both younger and older children associated African–American targets with indicators of low economic status more frequently than European–American targets. Finally, children associated African–American targets with indicators of high economic status less frequently with age, resulting in a perceived disparity in favor of European–American targets at high economic status among older children that was not present among younger children. No differences were found by participants’ own racial or ethnic background. These results highlight the need to move beyond a dichotomized view (rich or poor) to include middle economic status when examining children’s associations of economic resources and race.

## Introduction

Research in developmental science indicates that children are aware of which groups in their society, including racial groups, are considered high status (most respected or esteemed) beginning in early childhood (Nesdale, 2004; Brown and Bigler, 2005; Rutland et al., 2010). Likewise, with age, children are increasingly aware that individuals possess different amounts of resources, including economic resources (Horwitz et al., 2014; Mistry et al., 2015). Less research, however, has focused on children’s developing awareness of which groups possess economic resources. In this study, we examined age-related changes in children’s associations of economic resources and race.

Determining whether and when children perceive differences in the representation of racial groups at high, middle, and low levels of the economic spectrum is important for understanding children’s developing social and moral knowledge about status and social inequality. From an early age, children’s moral judgments involve notions of equal and fair treatment of others (Killen and Smetana, 2015). Most of the research on developing conceptions of *un*fair treatment, including knowledge about societal inequalities, however, has focused on adolescence, examining conceptions of fair government and freedoms (Helwig et al., 2014), explanations for wealth and poverty (Flanagan et al., 2014), and perceptions of how wealth is distributed in the United States (Arsenio et al., 2013). Despite a longstanding interest in fairness and distributive justice in childhood (Piaget, 1932; Damon, 1975), very little is known about what younger children think about broader social inequality.

One way to study children’s perceptions of social inequality is to measure whether children associate different levels of economic status with different groups, including racial groups. In many contexts, children scrutinize unequal social norms and institutions, and reject some as unjust (Wainryb et al., 2008). Yet, in order to be able to reject an unfair social structure, children must first recognize its existence. Indeed, there is little evidence that adopting a “color-blind” approach sufficiently prepares children to combat issues of inequality (Hughes, 2003; Levy et al., 2005; Pahlke et al., 2012; Elenbaas and Killen, in press). Age-related increases in children’s perceptions of racial groups’ differential representation on the economic spectrum reflect increases in awareness of social inequality. Thus, the primary aim of this study was to identify whether and when children perceive differential representation of racial groups at high, middle, and low levels of the economic spectrum.

### Children’s Awareness of Inequality

Interestingly, most developmental research with U.S. samples has conceptualized economic status on a dichotomized scale, assessing children’s knowledge of wealth and poverty, for example. Previous research indicates that, from as early as 4–5 years of age, children categorize individuals as rich or poor based on external, observable characteristics like house size or clothing quality (Ramsey, 1991; Weinger, 2000; Horwitz et al., 2014). Children’s conceptions of economic resources expand to include adult’s occupational status and factors like family connections and inheritance by at least 10–12 years of age (Chafel and Neitzel, 2005; Mistry et al., 2015).

However, most children in the U.S. come from backgrounds that are neither rich nor poor, but middle–income (DeNavas-Walt and Proctor, 2014). This distinction between a rich/poor dichotomy versus a wider economic scale is also important with respect to associations of race and economic status. Not long after they begin to identify economic differences between individuals, children begin to recognize correlations between race and economic resources. For example, one study found that 6–7 and 11–12 year-old African–American children were aware of the limited representation of African–American adults in high income occupations (e.g., doctor, business executive) (Bigler et al., 2003). Likewise, another recent study found that 7–11 year-old European–American children more readily associated European–Americans than African–Americans with expensive possessions (e.g., sports car, expensive video game console), but African–American children did not show the same pattern of associations (Newheiser and Olson, 2012).

Early associations of economic indicators with these racial groups may be the first step in children’s understanding of the larger, systemic imbalance of economic resources in the United States. In fact, some of the most striking group-level economic differences in the United States exist between African–Americans and European–Americans (Saegert et al., 2007). Yet, progress since the 1960s has witnessed the emergence of a large African–American middle class (Collins, 1983). Assessing children’s *relative* placement of African–Americans and European–Americans on the economic spectrum (which group is higher?) may mask important complexities and age-related changes in children’s associations of racial groups and possession of economic resources.

Further, while these studies represent important progress, very little is known about how children’s associations of economic resources with racial groups change with age, as no previous studies in this area have revealed age-related changes. This may be attributable to previous research questions and study designs, which focused on children’s relative placement of African–Americans and European–Americans on the two ends of the economic spectrum, potentially masking age-related changes in children’s understanding of groups’ differential representation a high, middle, and low levels of economic status. Indeed, children’s associations of race and economic status should change with age as they develop distinct notions about the structure of their society and social world (Wainryb et al., 2008).

Finally, one additional challenge in interpreting previous research in this area involves differences (or lack thereof) in African–American and European–American children’s associations of economic resources with race. This challenge arises from sampling differences between studies (racially diverse versus homogeneous samples), as well as methodological differences (e.g., implicit versus explicit associations). In order to clarify age-related changes in children’s associations of economic resources with African–Americans and European–Americans, a sample reflecting children of both racial backgrounds who share the same socioeconomic background is needed.

### The Current Study

The current study addressed the question: Do children differentially associate *high* levels, *middle* levels, and *low* levels of the economic spectrum with one racial group over another? If children do associate both African–Americans and European–Americans with middle levels of the economic spectrum at a comparable rate, showing differential associations only at the more extreme ends, then this would provide important evidence for their developing awareness of greater homogeneity at the highest and lowest points on the spectrum, and relative heterogeneity in the middle. Such evidence would contribute to the literature in this area by providing a more detailed account of children’s developing associations of economic resources and race, revealing when and children first recognize this foundational aspect of social inequality.

Thus, in this study, three levels of economic status –low, middle, and high– were measured in order to more accurately reflect what most children observe or experience in their everyday lives (Bradley and Corwyn, 2002; DeNavas-Walt and Proctor, 2014). Participants viewed pairs of target children who varied by race (African–American and European–American), and matched them to material possessions and future occupations indicative of wealth and income across a series of six matching trials. This array (high – middle – middle – low) allowed children to match individuals of both racial groups with middle levels of the economic spectrum, which comprises most of the U.S. population (DeNavas-Walt and Proctor, 2014).

In this study, we aimed to assess age-related changes in children’s perceptions during a time in development when conceptions of economic status become increasingly complex (as outlined above). Children ages 5–6 and 10–11 years old participated in the current study, spanning a time in development when children’s awareness of many cues to economic differences between individuals grows considerably (Ramsey, 1991; Weinger, 2000; Chafel and Neitzel, 2005; Horwitz et al., 2014; Mistry et al., 2015).

The sample included children of African–American and European–American background, as well as a sample of children not identified (by their parents) as African–American or European–American, in order to rule out the potential impact of ingroup bias in the interpretation of children’s responses. There are many cases in which children make positive associations with their racial ingroup over their racial outgroup (Rutland et al., 2005; Baron and Banaji, 2006; McGlothlin and Killen, 2006). Previous studies do not point to ingroup bias, however, as a factor in children’s associations of economic resources with racial groups. In fact, some research has shown that children’s tendency to associate higher-value material possessions with higher-status racial groups *cannot* be explained by a simple strategy of matching “positive” or appealing stimuli with preferred ingroups (Olson et al., 2012; Horwitz et al., 2014). Nevertheless, inclusion of children whose racial ingroup is not represented in the stimuli provides a measure of assurance, in that these children’s responses are unlikely to be swayed by potential motivation to associate their ingroup with higher levels economic status.

By presenting an economic spectrum that more closely resembled that of children’s typical peer environments, we provided participants with options that were not restricted to the highest and lowest ends of the economic spectrum. Accordingly, instead of comparing children’s overall associations for African–American and European–American targets relative to each other, our analyses focused on children’s perceptions of the relative representation of each group at each level (high, middle, and low economic status) separately.

### Hypotheses

The age groups chosen for this study (5–6 and 10–11 years) span a time of change in children’s awareness of cues to economic status (Ramsey, 1991; Weinger, 2000; Chafel and Neitzel, 2005; Horwitz et al., 2014; Mistry et al., 2015), and thus we predicted that children’s awareness of which racial groups are commonly represented at high, middle, and low levels of the economic spectrum would likewise increase with age. That is, we predicted that, between 5–6 and 10–11 years of age, children would increasingly associate targets of *both* races with indicators of middle economic status, reflecting their increasing awareness that the majority of the population occupies the middle of the economic spectrum. We also predicted, however, that, with age, children would be increasingly likely to associate European–American targets with indicators of higher economic status and increasingly likely to associate African–American targets with indicators of lower economic status, reflecting societal trends (Bigler et al., 2003; Newheiser and Olson, 2012).

We did not expect differences in associations by children’s own race. In fact, we expected that both African–American and European–American children would demonstrate the same age-related changes in patterns of association of race and economic resources. Previous research indicating that both African–American (Bigler et al., 2003) and European–American (Newheiser and Olson, 2012) children perceive racial disparities in access to economic resources supports this hypothesis. Inclusion of children from non-African–American/European–American background provided an additional check of this hypothesis, as these children’s responses should not be influenced by ingroup preference.

## Materials and Methods

### Participants

The total sample consisted of *N* = 308 children, including 153 5–6 year-olds (*M* = 6.01 years, *SD* = 0.33 years) and 155 10–11 year-olds (*M* = 11.12 years, *SD* = 0.59 years). The sample was relatively evenly divided by gender: 79 males and 73 females aged 5–6 years; 70 males and 85 females aged 10–11 years. Participant race/ethnicity was obtained by parent report. The distribution for 5–6 year-olds was: 31% (*n* = 48) European–American, 28% (*n* = 43) African–American, 21% (*n* = 32) Latino (not in combination with any other racial group), 6% (*n* = 10) Asian–American, 11% (*n* = 17) multi-racial or multi-ethnic, and 2% (*n* = 3) whose parents declined to provide race or ethnicity information. The distribution for 10–11 year-olds was: 28% (*n* = 44) European–American, 32% (*n* = 50) African–American, 19% (*n* = 30) Latino (not in combination with any other racial group), 8% (*n* = 13) Asian–American, 6% (*n* = 9) multi-racial or multi-ethnic, and 6% (*n* = 9) whose parents declined to provide race or ethnicity information.

Participants were recruited from eight racially/ethnically diverse elementary schools serving the same socioeconomic communities: middle- to lower-middle-income families in the same geographical area (Mid-Atlantic region) of the United States. Although no information on individual participant parental educational attainment or income level was available, across all schools, the percentage of children eligible for the FARMS (Free and Reduced Meals) program ranged from approximately 20% to approximately 70%. FARMS eligibility is calculated based on a combination of household size and income. Across all schools, the racial composition of the school population ranged from approximately 20% to approximately 40% African–American students and approximately 10% to approximately 50% European–American students. The average response rate across schools was approximately 70%. Written parental consent and children’s verbal assent were obtained for all participants. This project was approved by the Institutional Review Board at the University of Maryland.

### Procedure

Older children completed measures independently, while younger children were interviewed by a trained experimenter in a quiet space at their school. All stimuli and measures were presented using MediaLab v2012 (Empirisoft Corporation) on laptop computers. Study administration took approximately 15 minutes.

### Measures

Participants completed six trials associating targets to material possessions (three trials) and occupations (three trials). All material possessions (houses, cars, children’s bedrooms) were based on previous research on children’s associations of material possessions with wealth (e.g., Horwitz et al., 2014), and all occupations (airline pilot, electrician, mail carrier, fast food worker, scientist, police officer, teacher, janitor, doctor, office worker, bus driver, cashier) were based previous research on children’s judgments of different occupations with respect to income (Bigler et al., 2003). Pilot testing was also conducted with a small (*n* = 20) adult sample prior to data collection, in order to confirm that each image representing a material possession or an occupation was considered representative of high, middle, or low economic status.

For each trial, participants matched a European–American target and an African–American target to one of a set of four cues to economic resources. Each set of four cues to economic resources consisted of one higher option, two middle options, and one lower option (high – middle – middle – low array). That is, participants viewed pairs of target children who varied by race (African–American and European–American), and matched them to material possessions and future occupations indicative of wealth and income across a series of six matching trials.

In each trial, four images of material possessions or four icons representing occupations (with no human figures) appeared on the screen, accompanied by an introductory sentence (e.g., “Here are four houses,” or “Here are four jobs that people have”). Then two photographs of children, one African–American and one European–American, appeared on the screen, with an introductory sentence (e.g., “This kid lives in one of these houses, and this kid lives in one of these houses,” or “When this kid grows up, she/he will have one of these jobs, and when this kid grows up, she/he will have one of these jobs”).

Children were asked to match targets to material possessions or occupations one at a time. For example, in the ‘cars’ trial, children first saw four cars appear on the screen (one representing low economic status, two representing middle economic status, and one representing high economic status). Then they saw one African–American child and one European–American child appear on the screen. Children were told “This kid rides in one of these cars, and this kid rides in one of these cars.” Then, children were asked “Which car does this kid ride in?” in regards to the first target child. Participants matched the first target child with a car by pointing to or clicking a button, and the process was repeated for the other target child.

Targets could not both be matched to the same material possession or occupation. Half the sample matched African–American targets first, and half of the sample matched European–American targets first. In each trial targets were the same gender. Trials alternated between occupations and material possessions, and target gender alternated between trials.

### Data Coding

The frequency with which participants associated African–American targets and European–American targets with high, middle, and low economic status indicators was calculated across the materials possessions and occupations trials. Each frequency could range from 0 (never assigned targets of that race to that level) to 6 (always assigned targets of that race to that level).

Importantly, instead of comparing children’s overall associations for African–American and European–American targets relative to each other (as has been the focus of most previous work using dichotomous high/low scales), our hypotheses focused on children’s perceptions of the relative representation of each racial group at each level –high, middle, and low economic status–separately. For example, a participant who associated European–American targets with indicators of high economic status twice over the course of the six trials and African–American targets with indicators of high economic status once over the course of six trials would receive a score of 2 for associations of European–American targets with indicators of high economic status and a score of 1 for associations of African–American targets with indicators of high economic status. Our hypotheses and analyses focused on comparing how these scores (“2” and “1,” in this example) differed (or not) from each other within one level of the economic spectrum (in this case the “high” level) and not on how they might differ from scores for targets of the same race at another level. In short, our data were coded so that analyses could focus on children’s perceptions of the relative representation of each group at each level (high, middle, and low economic status) separately.

By design, the sample was primarily comprised of children of African–American (*n* = 93) and European–American (*n* = 92) background, however, children of other racial backgrounds (*n* = 123) were also recruited, in order to test our hypothesis that children of all backgrounds would demonstrate the same age-related changes in patterns of association of race and economic resources (i.e., differences in associations do not reflect ingroup bias). Thus, in the analyses presented below, participant race was split to form three categories: European–American, African–American, and Other Backgrounds (children whose parents identified them as Latino, Asian–American, multi-racial/multi-ethnic, or did not provide race/ethnicity information). Notably, the pattern of results does not change if the responses of multi-racial/multi-ethnic children and children whose parents did not provide race/ethnicity information are omitted from the sample.

## Results

### Preliminary Analyses

First we conducted preliminary analyses to confirm that children’s associations of race with indicators of high, middle, and low economic status did not differ significantly by participant gender, target gender, or the interaction of participant gender and target gender. There were no significant effects of these variables. Second, we conducted preliminary analyses to determine whether children’s associations of race with indicators of high, middle, and low economic status differed by indicator type (material possessions versus occupations). These analyses revealed no clear pattern of different results for different economic indicator types. Thus, we proceeded on to test our central hypotheses regarding children’s associations of race with economic status.

### Primary Analyses: Associations of Race and Economic Status Change with Age

We conducted a 2 (Age Group: 5–6 years, 10–11 years) × 3 (Participant Race: African–American, European–American, Other Backgrounds) × 2 (Target Race: European–American, African–American) × 3 (Economic Status: High, Middle, Low) ANOVA with repeated measures on the last two factors. That is, we assessed whether participant age group and race predicted differences in children’s associations of targets of different races (African–American and European–American) with indicators of high, middle, and low economic status.

This model revealed a main effect of Economic Status, *F*(2,604) = 516.07, *p* < 0.001, $\eta_{\text{p}}^{2}=0.63$, an interaction effect for Economic Status and Age Group, *F*(2,604) = 53.36, *p* < 0.001, $\eta_{\text{p}}^{2}=0.15$, an additional interaction effect for Economic Status and Target Race, *F*(2,604) = 22.17, *p* < 0.001, $\eta_{\text{p}}^{2}=0.07$, and a final interaction effect for Economic Status, Age Group, and Target Race, *F*(2,604) = 6.30, *p* = 0.002, $\eta_{\text{p}}^{2}=0.02$. There were no main or interaction effects for Participant Race.

The main effect for Economic Status indicated that, on average, children associated targets with middle levels (*M* = 3.48, *SE* = 0.06) more frequently than with low (*M* = 0.87, *SE* = 0.04) or high (*M* = 1.65, *SE* = 0.04) levels (as might be expected given the stimuli design), and associated targets with high levels more frequently than with low levels, all *ps* < 0.001.

Building on this main effect, the interaction for Economic Status and Age Group indicated that, while the overall frequency with which children associated targets with levels of economic status held for both younger children and older children (all *ps* < 0.001), frequency of associations at each separate level differed with age. Relative to younger children, older children associated targets with low levels less frequently (*M_Older_* = 0.59, *SE* = 0.06 versus *M_Y ounger_* = 1.14, *SE* = 0.06), associated targets with middle levels more frequently (*M_Older_* = 3.98, *SE* = 0.08 versus *M_Y ounger_* = 2.98, *SE* = 0.09), and associated targets with high levels less frequently (*M_Older_* = 1.43, *SE* = 0.06 versus *M_Y ounger_* = 1.87, *SE* = 0.06); all *ps* < 0.001.

Additionally, the second interaction effect, Economic Status × Target Race, revealed that, overall, children associated African–American targets with low levels of economic status (*M* = 1.14, *SD* = 1.14) more frequently than European–American targets (*M* = 0.59, *SD* = 0.90), *p* < 0.001, and associated European–American targets with high levels of economic status (*M* = 1.94, *SD* = 1.28) more frequently than African–American targets (*M* = 1.36, *SD* = 1.15), *p* < 0.001. Associations of targets with middle economic status did not differ significantly by target race (*M_Af_*_-_*_Am_* = 3.49, *SD* = 1.50 and *M_Eu_*_-_*_Am_* = 3.47, *SD* = 1.43), *p* = 0.81. Further, although children associated targets of both races with middle economic status more frequently than with high or low economic status (all *ps* < 0.001), children did not differ significantly in the extent to which they associated African–American targets with low versus high economic status, *p* = 0.08, whereas they associated European–American targets with high economic status significantly more frequently than with low economic status, *p* < 0.001.

**FIGURE 1:**
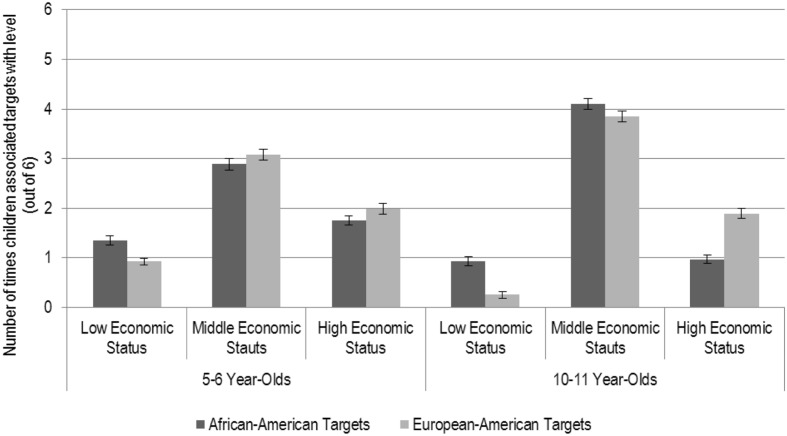
**Five to six year-olds’ and ten to eleven year-olds’ associations of African–American and European–American targets with low, middle, and high economic status.** Bars represent the standard error of the mean.

The final interaction for Economic Status, Age Group, and Target Race is illustrated in **Figure 1.** First, in line with our prediction, with age, children increasingly associated targets (of both races) with middle economic status (both *ps* < 0.001). Further, neither younger (*p* = 0.19) nor older (*p* = 0.09) children significantly associated target race with middle economic status; for younger children *M_Af_*_-_*_Am_* = 2.89, *SD* = 1.25 and *M_Eu_*_-_*_Am_* = 3.08, *SD* = 1.29; for older children *M_Af_*_-_*_Am_* = 4.10, *SD* = 1.48 and *M_Eu_*_-_*_Am_* = 3.85, *SD* = 1.46. Thus, children associated targets of both races with middle economic status at a comparable rate.

Next, although children associated targets (of both races) with low economic status less frequently at 10–11 years than at 5–6 years (*p* = 0.001 for African–American targets and *p* < 0.001 for European–American targets), both younger children and older children still associated African–American targets (*M_Y ounger_* = 1.36, *SD* = 0.95, *M_Older_* = 0.93, *SD* = 1.26) with low economic status more frequently than European–American targets (*M_Y ounger_* = 0.93, *SD* = 0.91, *M_Older_* = 0.26, *SD* = 0.74), both *ps* < 0.001. Thus, both younger and older children associated African–American targets with low economic status more frequently than European–American targets.

Additionally, children associated African–American targets with high economic status less frequently at 10–11 years (*M* = 0.97, *SD* = 1.06) than at 5–6 years (*M* = 1.76, *SD* = 1.11), *p* < 0.001, but associations of European–American targets with high economic status did not change significantly with age (*M_Y ounger_* = 1.99, *SD* = 1.19 and *M_Older_* = 1.89, *SD* = 1.35), *p* = 0.5. This resulted in a significant disparity in favor of European–Americans at high economic status for older children (*p* < 0.001) that was not present for younger children (*p* = 0.12). That is, while younger children associated targets of both races with high economic status at a comparable rate, older children associated European–American targets with high economic status more frequently than African–American targets.

Finally, although our hypotheses focused on associations at each level (low, middle, and high) individually (as presented above), for both younger and older children, the main findings for relative placement held, in that children more frequently associated targets of both races with middle economic status than with low or high economic status (all *ps* < 0.001). Additionally, for European–American targets, frequency of association with high economic status was greater than with low economic status among younger and older children (all *ps* < 0.001). For African–American targets, frequency of association with high economic status was greater than with low economic status among younger children (*p* = 0.02) but did not differ significantly among older children (*p* = 1.0).

In short: (1) children associated targets of both races with middle economic status at a comparable rate and, with age, increasingly associated targets of both races with middle economic status; (2) children associated African–American targets with low economic status more frequently than European–American targets; (3) with age, children associated African–American targets with high economic status less frequently, resulting in a perceived disparity in favor of European–American targets at high economic status for older children that was not present for younger children.

### Additional Analyses

As supplementary tests, following our primary analyses (reported above), we also compared the frequencies with which younger and older children associated African–American and European–American targets with each level of economic status against the rate that would be expected by chance. The high and low levels each comprised 25% of possible “match” options across six trials, thus chance frequency for high and low levels was 1.5. The middle level comprised 50% of possible “match” options across six trials, thus chance frequency was 3.

For middle economic status, younger children associated both African–American (*M* = 2.89, *SD* = 1.25) and European–American (*M* = 3.08, *SD* = 1.29) targets at rates that did not differ significantly from chance; *t*(152) = –1.16, *p* = 0.25, *d* = 0.19 and *t*(152) = 0.69, *p* = 0.49, *d* = 0.11. Older children, however, associated both African–American (*M* = 4.10, *SD* = 1.48) and European–American (*M* = 3.85, *SD* = 1.46) targets with middle economic status more frequently than would be expected by chance; *t*(154) = 9.45, *p* < 0.001, *d* = 1.52 and *t*(154) = 7.39, *p* < 0.001, *d* = 1.19. For low economic status, both younger (*M* = 0.93, *SD* = 0.91) and older (*M* = 0.26, *SD* = 0.74) children associated European–American targets less frequently than would be expected by chance; *t*(152) = –7.64, *p* < 0.001, *d* = –1.24 and *t*(154) = –20.81, *p* < 0.001, *d* = –3.35. Further, older children (*M* = 0.93, *SD* = 1.26), but not younger children (*M* = 1.36, *SD* = 0.95) associated African–American targets with low economic status less frequently than would be expected by chance; *t*(152) = –1.82, *p* = 0.07, *d* = –0.29 and *t*(154) = –5.75, *p* < 0.001, *d* = –0.93. For high economic status, both younger (*M* = 1.99, *SD* = 1.19) and older (*M* = 1.89, *SD* = 1.35) children associated European–American targets more frequently than would be expected by chance; *t*(152) = 5.11, *p* < 0.001, *d* = 0.83 and *t*(154) = 3.42, *p* = 0.001, *d* = 0.55. However, while younger children (*M* = 1.76, *SD* = 1.11) associated African–American targets with high economic status more frequently than would be expected by chance, older children (*M* = 0.97, *SD* = 1.06) associated African–American targets with high economic status less frequently than would be expected by chance; *t*(152) = 2.89, *p* = 0.004, *d* = 0.47 and *t*(154) = –6.31, *p* < 0.001, *d* = 1.02.

## Discussion

The novel findings of this study revealed that children’s associations of economic resources with racial groups changed with age, and reflected different associations at high, middle, and low levels of the economic spectrum (in the United States context). Interestingly, 5–6 year-olds’ associations reflected the over-representation of African–Americans at lower levels of the economic spectrum, but showed no significant differences in associations by race at higher levels. At 10–11 years, however, children’s associations reflected *both* the over-representation of African–Americans at lower levels, *and* the over-representation of European–Americans at the higher end of the economic spectrum. Further, with age, children increasingly associated targets of both races with middle economic status, reflecting societal trends.

This study makes two primary contributions to the literature in this area. First, these findings have important implications for understanding social and moral development in early and middle childhood. Despite a long standing interest and growing research focus on distributive justice in childhood, very little research has examined children’s perceptions of broader social inequalities. Findings from this study indicate that children are aware of racial groups’ differential representation at different levels of the economic spectrum from as early as 5–6 years of age, and this awareness of social inequality increases with age. Thus, the reasoning about groups’ rights, structural causes of poverty, and fair distribution of wealth that emerges later in adolescence (e.g., Arsenio et al., 2013; Flanagan et al., 2014; Helwig et al., 2014) may be built on the foundation of these early associations reflecting awareness of social inequality. This study represents an important step toward understanding younger children’s thinking about group-level representation on the economic spectrum, in addition to judgments about the equal treatment of individuals.

The second major contribution of this study pertains to the new knowledge about children’s awareness of inequality reflected in our assessment of children’s associations of race and economic resources separately at high, middle, and low levels of the economic spectrum. These findings revealed a more complex picture of children’s awareness of inequality than that represented in the literature to date, which has focused on children’s relative placement of African–Americans and European–Americans on the economic spectrum. This broader approach revealed that 5–6 year-olds perceived an over-representation of African–Americans at the low end of the economic spectrum, and the magnitude of this perceived disparity did not change significantly with age. What changed with age were children’s perceptions of the middle and high levels of the economic spectrum. Specifically, unlike their younger counterparts, 10–11 year-olds were also aware of the over-representation of European–Americans at the higher end of the economic spectrum.

Thus, supporting our hypotheses, with age, children’s associations of economic resources with race increasingly reflected societal trends. In line with research on children’s broader understanding of economic inequalities between individuals (Ramsey, 1991; Weinger, 2000; Chafel and Neitzel, 2005; Horwitz et al., 2014; Mistry et al., 2015), these findings emphasize the early emergence and continuing development of children’s associations of race and economic resources. Also supporting our hypotheses, with age, children increasingly associated targets of both racial groups with the middle of the economic spectrum, reflecting their increasing awareness that members of either racial group could occupy middle levels of economic status.

These findings about middle economic status are equally as noteworthy as children’s associations at the extremes of the economic spectrum, as they provide evidence for developing awareness of greater homogeneity at the highest and lowest points on the economic spectrum combined with relative heterogeneity in the middle. These findings could be interpreted as revealing a more positive picture than that represented in the literature to date: children perceive that both African–Americans and European–Americans are likely to occupy the middle of the economic spectrum, and these perceptions increase with age. The relatively low rate of overall associations with low economic status (with the inclusion of a middle-status option) is also positive. Inclusion of a middle-status option, in contrast to measures of relative placement, revealed important nuances in children’s perceptions that provide a more detailed reflection of their everyday observations and experiences.

Somewhat surprisingly, we found no significant differences in children’s associations of race and economic resources by participants’ own racial or ethnic background. That is, whereas extensive research has demonstrated children’s positive associations with their own racial group (ingroup bias), findings from this study revealed a consistent pattern of age-related changes in children’s associations of race and economic resources with a racially/ethnically diverse sample of children from middle- to low-middle income backgrounds. Research on children’s ingroup biases is important for understanding intergroup relations in development, yet this study suggests that children’s associations of race and economic resources follow a different developmental trajectory from their positive associations with their racial ingroup (see also Olson et al., 2012; Horwitz et al., 2014). Rather than associating their racial ingroup with higher economic status, this study found that, with age, children made associations of race and economic resources that increasingly reflected perceived disparities at the lower, middle, and higher positions on the economic spectrum, irrespective of their own group membership. These results provide support for the notion that children from different racial and ethnic backgrounds recognize the same status hierarchies that exist in the U.S. social context concerning access to economic resources.

### Limitations and Future Directions

This study included a racially/ethnically diverse sample of participants in order to examine age-related changes while accounting for potential ingroup biases in children’s responses. Participants were, however, relatively homogenous in socioeconomic status. Thus, an important next step for this research is to investigate the impact of children’s own position on the economic spectrum, and that of their peers, on age-related changes in their associations of economic resources with racial groups. Related work on children’s understanding of economic disparities between individuals suggests that, with age, children’s own socioeconomic status, or personal experiences interacting with individuals of varying socioeconomic status, may predict some variability in their broader understanding of the causes of economic inequality (Chafel and Neitzel, 2005; Flanagan et al., 2014), which may also include racial biases as a cause. Thus, an important next step for future research is to examine the joint and separate effects of participant race and participant SES (as well as the race and SES of their peers at school) on age-related changes in children’s associations of racial groups with economic resources at high, middle, and low levels of the economic spectrum. Assessment of children’s associations of economic resources with racial/ethnic groups other than those represented in this study (e.g., Asian–Americans, Latinos) will also be important for gaining a complete picture of children’s perceptions regarding the distribution of economic resources in society.

Further, the high – middle – middle – low economic spectrum used in the current study was designed to provide a more ecologically valid test of children’s associations of race and economic resources by allowing children to match individuals of both racial groups with middle levels of the economic spectrum, which comprises most of the U.S. population. While this design revealed age-related changes in children’s conceptions of inequality at three levels of the economic spectrum, one limitation of this approach is that, on a given trial, children could not associate both targets with high or with low economic status. Future studies in this area could consider providing two options at each of the three levels in order to further test children’s associations at the high and low ends of the economic spectrum, and provide a means for relative comparison of average associations for one racial group versus another.

Future research should also explore the relation between children’s associations of race and economic resources and their endorsement or rejection of such disparities. Although children associate race and economic resources, this does not necessarily mean that they believe such disparities are fair or legitimate. In many contexts, children evaluate social norms and institutions, and reject some as unjust (Wainryb et al., 2008). Thus, a natural next step for this line of work is to determine the extent to which children judge economic inequalities linked with race to be fair or unfair. In fact, emerging research suggests that awareness of larger economic disparities between groups may motivate children to alleviate resource inequalities (Killen et al., 2016; Elenbaas and Killen, in press). Thus, more research is needed in order to clarify how children’s developing associations of race and economic resources relate to their judgments about the acceptability (or unacceptability) of economic disparities.

## Conclusion

This study provided evidence that children’s associations of economic resources with racial groups change with age, and reflect different associations at higher, middle, and lower levels of the economic spectrum. Interestingly, 5–6 year-olds’ associations reflected the over-representation of African–Americans at lower levels of the economic spectrum, but it was not until 10–11 years that children’s associations reflected the over-representation of European–Americans at higher levels of the economic spectrum. Importantly, with age, children increasingly associated targets of both races with middle levels of the economic spectrum.

These findings have implications for research on children’s social and moral development, and emerging awareness of social inequality, as outlined above. Further, from an applied perspective, information about how children represent the economic status of African–Americans and European–Americans is also vital for designing effective educational programs to expand individuals’ economic opportunities. One study, for example, found that a workshop designed help children identify biases in the workplace and develop strategies to counteract them increased 10–13 year-old African–American children’s expectations for the status of the job that they would one day have (Hughes, 2011). Thus, this basic research on age-related changes in children’s perceptions of differences in the economic status of African–Americans and European–Americans can inform both theoretical and applied advances in developmental science.

## Author Contributions

Both LE and MK made substantial contributions to: the conception and design of the work; the acquisition, analysis, and interpretation of data; and drafting the work and revising it critically for important intellectual content. Both LE and MK gave final approval of the version to be published, and agree to be accountable for all aspects of the work.

## Conflict of Interest Statement

The authors declare that the research was conducted in the absence of any commercial or financial relationships that could be construed as a potential conflict of interest.
